# Effects of two types of *Coccomyxa* sp. KJ on *in vitro* ruminal fermentation, methane production, and the rumen microbiota

**DOI:** 10.1371/journal.pone.0308646

**Published:** 2024-08-22

**Authors:** Yoshiaki Sato, Honoka Shioya, Yuma Uda, Hiroshi Asano, Yoshikazu Nagao, Hitoshi Kuno, Fumiaki Yoshizawa

**Affiliations:** 1 Department of Agrobiology and Bioresources, School of Agriculture, Utsunomiya University, Tochigi, Japan; 2 University Farm, School of Agriculture, Utsunomiya University, Tochigi, Japan; 3 KJ Bio Co., Ltd., Kumamoto, Japan; Zagazig University Faculty of Agriculture, EGYPT

## Abstract

*Coccomyxa* sp. KJ is a unicellular green microalga that accumulates abundant lipids when cultured under nitrogen-deficient conditions (KJ1) and high nitrogen levels when cultured under nitrogen-sufficient conditions (KJ2). Considering the different characteristics between KJ1 and KJ2, they are expected to have different effects on rumen fermentation. This study aimed to determine the effects of KJ1 and KJ2 on *in vitro* ruminal fermentation, digestibility, CH_4_ production, and the ruminal microbiome as corn silage substrate condition. Five treatments were evaluated: substrate only (CON) and CON + 0.5% dry matter (DM) KJ1 (KJ1_L), 1.0% DM KJ1 (KJ1_H), 0.5% DM KJ2 (KJ2_L), and 1.0% DM KJ2 (KJ2_H). DM degradability-adjusted CH_4_ production was inhibited by 48.4 and 40.8% in KJ2_L and KJ2_H, respectively, compared with CON. The proportion of propionate was higher in the KJ1 treatments than the CON treatment and showed further increases in the KJ2 treatments. The abundances of *Megasphaera*, *Succiniclasticum*, *Selenomonas*, and *Ruminobacter*, which are related to propionate production, were higher in KJ2_H than in CON. The results suggested that the rumen microbiome was modified by the addition of 0.5–1.0% DM KJ1 and KJ2, resulting in increased propionate and reduced CH_4_ production. In particular, the KJ2 treatments inhibited ruminal CH_4_ production more than the KJ1 treatments. These findings provide important information for inhibiting ruminal CH_4_ emissions, which is essential for increasing animal productivity and sustaining livestock production under future population growth.

## Introduction

The sustainability of livestock production is crucial because the demand for animal protein products, such as meat and milk, is increasing with global population growth. Ruminants play a pivotal role in supplying food to humans; however, they are also the primary emitters of methane (CH_4_), a greenhouse gas (GHG). CH_4_ emitted during ruminal fermentation accounts for 39% of the GHG emissions in the agricultural sector [1]. Additionally, CH_4_ emissions result in dietary energy losses ranging from 2 to 12% in ruminants [2]. Thus, inhibiting ruminal CH_4_ emissions is essential for increasing animal productivity and sustaining livestock production. Consequently, dietary strategies for mitigating ruminal CH_4_ using feed additives, such as synthetic compounds [3, 4], cashew byproducts [5], fats and fatty acids [6–9], and organic acids [10–12], have attracted substantial attention.

Microalgae are microscopic unicellular organisms that efficiently convert solar energy into valuable bioactive compounds, such as proteins, lipids, and carbohydrates, thus indicating their commercial potential to enhance the nutritional value of animal feed supplements [13]. For example, dietary *Spirulina platensis* increases the average daily gain in lambs [14] and milk yield in cows [15]. Microalgae have also attracted attention as feed additives to inhibit CH_4_ production in ruminants. Some microalgae are rich in n–3 polyunsaturated fatty acids (PUFA) and can inhibit methanogenesis in the rumen, thereby shifting volatile fatty acid (VFA) production from acetate to propionate [16]. In fact, several microalgae, such as *Euglena gracilis* [17] and *Chlorella vulgaris* [18], inhibit CH_4_ production during ruminal fermentation. However, microalgae can also cause adverse effects in ruminants. For example, dietary *Schizochytrium* sp. decreases milk yield in dairy cows [19], while *Nannochloropsis gaditana*, *Phaeodactylum tricornutum*, and *Schizochytrium* sp. do not have anti-methanogenic effects *in vitro* [20]. Thus, although microalgae can be used as feed additives for ruminants, the effects of microalgal supplementation are debatable and different types of microalgae have different effects on rumen fermentation, CH_4_ production, and animal productivity.

*Coccomyxa* sp. KJ (IPOD FERM BP-22254) is a unicellular green microalga isolated from hot springs in Japan and belongs to the class Trebouxiophyceae. *Coccomyxa* sp. KJ can grow under low-pH conditions (pH 3.0–4.0) [21]. Owing to these characteristics, *Coccomyxa* sp. KJ can be cultivated in an open pond without contamination by other microorganisms and can be easily produced on an industrial scale. The KJ strain exhibits different characteristics when cultured under different conditions. For example, it accumulates lipids at > 30% of the dry cell weight when cultured under nitrogen-deficient conditions (KJ1) [21]. In contrast, the KJ strain contains high amounts of nitrogen when cultured under nitrogen-sufficient conditions (KJ2). KJ1 can be used in biofuel production [22], whereas KJ2 and its components can be used as immune-promoting supplements [23] and antiviral agents [24–27]. In ruminant feeds, KJ1 and KJ2 represent promising supplements as fat and nitrogen sources. In particular, because KJ2 contains a high amount of linolenic acid [23], using it as a feed additive for ruminants could change the fatty acid composition of milk and meat, thereby improving the product quality. In addition, supplementation with *Coccomyxa* sp. KJ is expected to change the rumen microbiome, resulting in the inhibition of CH_4_ production by the high amount of long-chain PUFAs [23], thus, this microalga has promising potential as a CH_4_ inhibitor [7–9].

Considering the differences in the characteristics between KJ1 and KJ2, the effects of these two types of *Coccomyxa* sp. KJ on rumen fermentation are expected to differ. However, previous studies have not investigated these differences. Furthermore, previous studies have not reported on the use of *Coccomyxa* sp. KJ as a feed additive for ruminants. Thus, the appropriate amount to use an additive must be determined. Therefore, this study aimed to determine the appropriate amount of supplementary *Coccomyxa* sp. KJ and investigate the effects of KJ1 and KJ2 as additives to corn silage substrate, which is commonly used for dairy production in Japan and worldwide, on *in vitro* ruminal fermentation, digestibility, CH_4_ production, and the ruminal microbiome.

## Materials and methods

### Ethical approval

The study was approved by the Utsunomiya University Animal Ethics Committee (approval no. A22-0013). Anesthesia and euthanasia were not performed in this study.

### Substrate, additives, and experimental treatments

**Fig 1:**
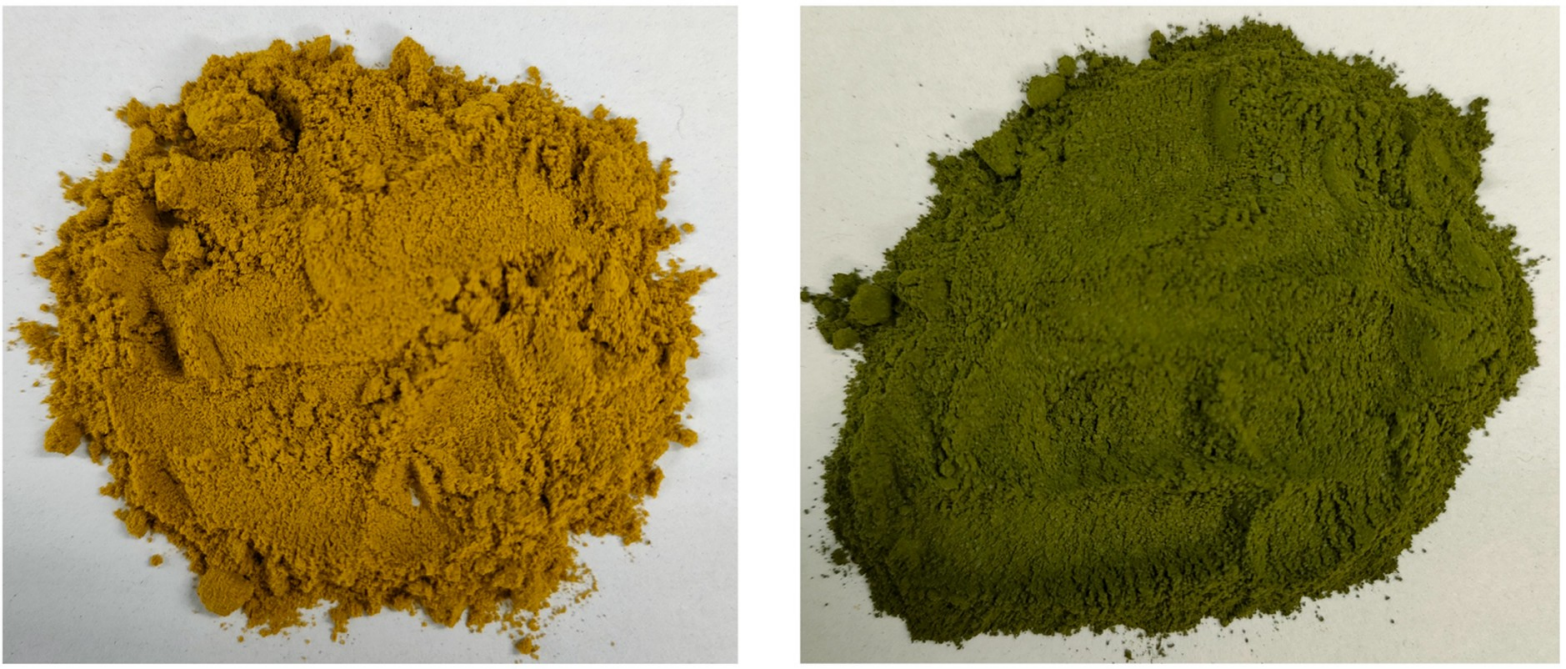
Images of dried powders of two types of *Coccomyxa* sp. KJ. The images on the left and right show KJ1 and KJ2, respectively.

Collected corn silage was dried at 60°C, ground using a sanitary crusher (SC-02, Sansho Industry Co. Ltd, Osaka, Japan), and passed through a 1 mm screen. The sample was used as the substrate for *in vitro* incubation. KJ1 and KJ2 were separately incubated in open ponds, concentrated by centrifugation, and dried to a powder at 140°C using a drum dryer. The dried KJ1 and KJ2 powders were used as additives (Fig 1). The following five experimental treatments were applied: I) substrate only (CON), II) CON + 0.5% dry matter (DM) KJ1 (KJ1_L), III) CON + 1.0% DM KJ1 (KJ1_H), IV) CON + 0.5% DM KJ2 (KJ2_L), and V) CON + 1.0% DM KJ2 (KJ2_H).

### *In vitro* experiments

Two female Holstein cows (body weight: 593 ± 63.6 kg, parity: 3 and 4) at the Utsunomiya University Farm were used. The animals were mostly housed in a tie-stall housing system, although they were allowed to graze on Italian ryegrass-based pasture from 09:00 to 13:00. The cows were primarily fed corn silage and concentrate five times daily at 05:30, 07:00, 13:00, 17:00, and 21:00. The ingredient compositions of the concentrate were as follows: 28.6% corn, 24.2% soybean meal, 13.2% barley, 8.8% wheat bran, 8.2% rice bran, 6.6% cotton seed, 5.5% fodder beet, 2.7% CaCO_3_, 1.1% NaCl, and 1.1% vitamin-mineral premix on a fresh matter basis. The average 7-day feed intake of corn silage and concentrate before sampling was 7.7 ± 0.14 kg and 7.4 ± 0.35 kg on a DM basis, respectively. Additionally, timothy hay was offered at < 2.5 kg daily. The cows were provided *ad libitum* access to water.

Rumen liquid (approximately 200 mL) was collected from each animal through orogastric tubing before the first feeding. The rumen samples were strained through four layers of gauze and mixed equally. The mixed samples were placed in preheated collection bottles and immediately transferred to the laboratory within 30 min. The rumen sample and artificial saliva [28], which was flushed with CO_2_, were mixed at a ratio of 1:4. The mixture (40 mL) was infused into each test tube containing 0.5 gDM of the substrate and additives under a stream of CO_2_. All tubes were immediately closed with rubber stoppers fitted with a plastic syringe to collect fermentation gas. The tubes were then incubated for 24 h at 39°C. Each treatment and blank containing only the mixture was set up in triplicates. The cumulative gas production at 0, 3, 6, 9, 12, 18, and 24 h was recorded during incubation. After incubation, samples (0.5 mL) were collected for DNA extraction and stored at −80°C, and additional 0.5 mL of the culture was mixed with 4.5 mL of methyl green formalin saline (MFS) solution to count the number of protozoa [29]. The remaining samples were centrifuged at 500 × *g* for 5 min. Subsequently, the pH was measured (LAQUAtwin pH-33B, HORIBA, Kyoto, Japan), and 10 mL of the supernatant was mixed with 2 mL of 25% metaphosphate solution to analyze the VFA and ammonia nitrogen (NH_3_-N) concentrations. The residue was used to determine DM degradability. The gas production, CH_4_ production, and DM degradability values for the experimental treatments were correlated with those of the blank.

### Chemical analyses

The substrate and additives were analyzed for DM, ether extract, and crude ash content according to the standards of the Association of Official Analytical Chemists (AOAC; 930.15, 920.39, and 942.05, respectively) [30]. The crude protein content was determined using the Dumas method with a nitrogen analyzer (Sumigraph NC-TRINITY; Sumika Chemical Analysis Service, Tokyo, Japan). The amylase-treated neutral and acid detergent fiber contents were determined as previously described [31]. The chemical compositions of the experimental feeds and substrates are shown in Tables 1 and 2, respectively. To measure DM degradability, the incubation residue was dried at 105°C until reaching a constant weight. To determine the fatty acid composition of *Coccomyxa* sp. KJ, direct transesterification was performed using a previously described method [32]. The fatty acid methyl ester (FAME) contents were analyzed using a gas chromatography system (GC-2010 Plus, Shimadzu Co., Ltd., Kyoto, Japan) equipped with a flame ionization detector (FID) and a capillary column (SP-2560, 100 m × 0.25 mm × 0.2μm, Supelco, Pennsylvania, USA) at a split rate of 100. The column, injector, and detector temperatures were 185°C, 250°C, and 250°C, respectively. The FAME contents were identified by matching the retention times with the standards of the Supelco® 37 Component FAME Mix. VFA concentrations were measured using gas chromatography (GC-2014, Shimadzu, Kyoto, Japan) equipped with a FID and a Restek Stabilwax column (30 m × 0.32 mm × 0.50 μm) at a split rate of 5. The temperatures of the injection and detector were 200°C and 250°C, respectively. The column temperature was linearly increased from 120°C to 230°C at 10°C/min. CH_4_ production was determined using a GC-2014 (Shimadzu) equipped with a FID and a capillary column (SH-Q-BOND, 30 m × 0.53 mm × 20 μm, Shimadzu) at a split rate of 5. The column, injection, and detector temperatures were 220°C, 250°C, and 250°C, respectively. The NH_3_-N concentration was analyzed using the microdiffusion method [33].

**Table 1:** Chemical composition of the feeds and *Coccomyxa* sp. KJ (% dry matter basis).

	Concentrate	Corn silage	KJ1	KJ2
Dry matter (%)	88.9	38.1	96.9	95.5
Crude protein	24.0	5.9	20.6	55.9
Ether extract	5.6	3.9	22.8	11.5
aNDF	22.8	42.1	23.4	10.2
ADF	9.1	25.1	8.1	2.4
Crude ash	8.6	4.9	1.7	4.7

aNDF, amylase-treated neutral detergent fiber; ADF, acid detergent fiber

**Table 2:** Chemical composition of the substrates in treatments (% dry matter basis).

Item^1^	CON	KJ1_L	KJ1_H	KJ2_L	KJ2_H
Crude protein	5.9	6.0	6.1	6.2	6.4
Ether extract	3.9	3.9	4.0	3.9	3.9
aNDF	42.1	42.0	41.9	41.9	41.7
ADF	25.1	25.0	25.0	25.0	24.9
Crude ash	4.9	4.9	4.9	4.9	4.9

^1^The values were calculated based on data in Table 1.

aNDF, amylase-treated neutral detergent fiber; ADF, acid detergent fiber

### DNA extraction, amplicon sequencing, and bioinformatics

The liquid samples after incubation were thawed and centrifuged at 12,000 × *g* at 4°C for 15 min. After removing the supernatants, the pellets were used for DNA extraction, as previously described [34] and slightly modified [35]. The extracted DNA was stored at -20°C until use.

To amplify prokaryotic DNA, the V3–V4 hypervariable region of the 16S rRNA genes was amplified using PCR with Pro341F (5′-CCTACGGGNBGCASCAG-3′) and Pro805R (5′-GACTACNVGGGTATCTAATCC-3′) primers [36]. Additionally, RP841F (5′-GACTAGGGATTGGARTGG-3′) and Reg1302R (5′-AATTGCAAAGATCTATCCC-3′) were used for protozoal 18S rRNA gene amplification [37]. Forward and reverse primers were tagged with the Illumina overhang adapter (forward: TCGTCGGCAGCGTCAGATGTGTATAAGAGACAG, reverse: GTCTCGTGGGCTCGGAGATGTGTATAAGAGACAG). Amplification was performed under the following conditions: 95°C for 3 min; followed by 25 or 35 cycles of 95°C for 30 s, 55°C for 30 s, and 72°C for 30 s for the prokaryotic and protozoal primers, respectively; and a final elongation step at 72°C for 5 min. The samples were indexed using a Nextera XT index kit (Illumina, San Diego, CA, USA) and paired-end sequenced on an Illumina MiSeq platform (2 × 300 bp).

After sequencing, the data were analyzed using QIIME2 [38]. Paired-end reads were trimmed and merged, and chimeric sequences were removed using the DADA2 plugin [39], followed by the construction of a feature table of amplicon sequence variants (ASVs). Taxonomy was assigned using the SILVA 138 reference database [40]. For the prokaryotic analysis, ASVs taxonomically assigned to the unassigned kingdom, eukaryotes, mitochondria, and chloroplasts were removed, whereas ASVs assigned to the unassigned kingdom, bacteria, and archaea were removed for protozoa. For diversity analysis, all sequence data were rarefied to the lowest sample depths of 32,879 and 1,737 sequences per sample for the prokaryotes and protozoa, respectively. The observed ASVs and Shannon diversity indices [41] were estimated using the ‘Phyloseq’ package of R [42]. The weighted UniFrac distance metric based on ASV was calculated using the ‘Phyloseq’ package [42], and the principal coordinates analysis (PCoA) plot was visualized with ‘ggplots2’ in R [43].

### Statistical analyses

The pH, gas and CH_4_ production, DM degradability, NH_3_-N content, VFA concentration, protozoal count data, and alpha diversity were analyzed using the GLM procedure in SAS Studio 9.04.01. The mathematical model was as follows:

$$Y_{ij}=\mu +T_{i}+e_{ij}$$

where *μ* represents the overall mean, *T*_*i*_ represents the effect of treatment, and *e*_*ij*_ represents the residual error. For beta diversity, permutational multivariate analysis of variance (PERMANOVA) with 9,999 permutations was performed. Differential abundance analysis between each group for microbial composition was performed using the Wald test within DESeq2 based on the read count matrix [44], and P-values were adjusted using the Benjamini–Hochberg method. Differences were considered statistically significant at P < 0.05.

## Results

### Fatty acid composition

The fatty acid composition of the *Coccomyxa* sp. KJ is shown in Table 3. The percentages of C16:0 (palmitic acid) in KJ1 and KJ2 were equivalent at 19.15 and 17.82%, respectively. The C18:1 (oleic acid) content in KJ1 was largely dominant at 56.67% and was approximately 2.7 times higher than that in KJ2 (20.87%). In contrast, PUFA such as C18:2 (linoleic acid) and C18:3 (linolenic acid) in KJ2 were 10.63 and 29.61%, respectively, and were more abundant compared to KJ1.

**Table 3:** Fatty acid composition (%) of *Coccomyxa* sp. KJ.

	KJ1	KJ2
C16:0	19.15	17.82
C18:0	3.56	0.78
*cis*-C18:1	56.67	20.87
*cis*-C18:2	5.78	10.63
*cis*-C18:3	11.13	29.61
C20:0	0.57	0.00
*cis*-C20:1	0.60	0.00
Others	2.56	20.29

### *In vitro* gas and CH_4_ production

**Fig 2:**
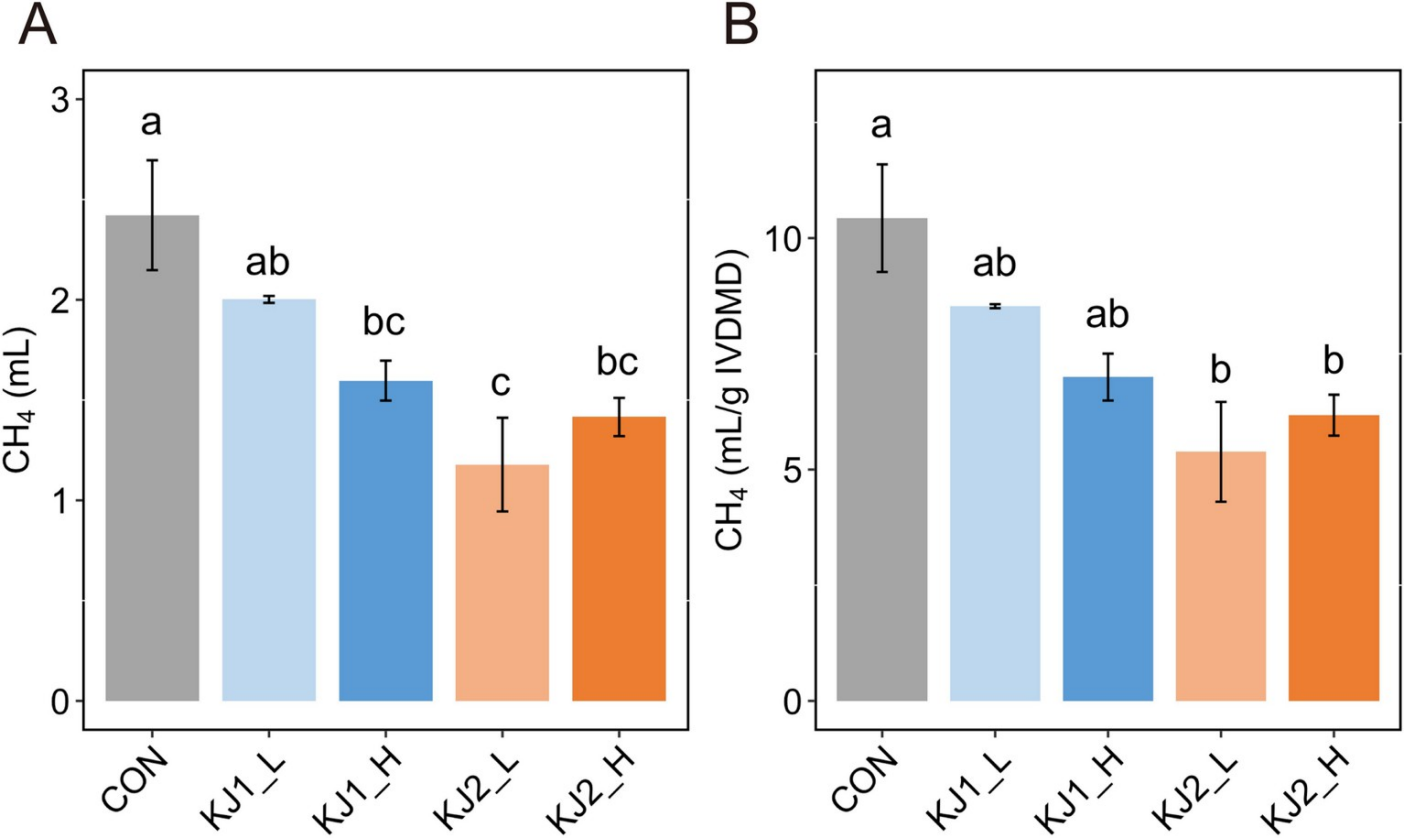
*In vitro* methane (CH_4_) production after 24 h of incubation. (A) Cumulative CH_4_ production and (B) dry matter degradability-adjusted CH_4._ Significant differences are indicated by different superscripts (P < 0.05). IVDMD, *in vitro* dry matter degradability.

Total gas production was decreased after 24 h of incubation in KJ1_H, KJ2_L, and KJ2_H (P < 0.05) compared to that in CON. In contrast, no significant differences were observed between the KJ1_L and CON treatments (Table 4 and S1 Fig). Similarly, total CH_4_ production in KJ1_H, KJ2_L, and KJ2_H was 34.1, 51.3, and 41.6% lower than that in CON (P < 0.05), respectively (Fig 2A). DM degradability-adjusted CH_4_ production was inhibited by 48.4 and 40.8% in KJ2_L and KJ2_H compared to that in CON (P < 0.05), respectively (Fig 2B).

**Table 4:** Effect of two types of *Coccomyxa* sp. KJ on *in vitro* rumen fermentation after 24 h incubation.

	Treatment					SEM	P value
	CON	KJ1_L	KJ1_H	KJ2_L	KJ2_H		
Gas production (mL/0.5gDM)	57.0^a^	56.2^a^	48.7^b^	40.6^c^	45.7^bc^	1.54	< 0.01
pH	6.87	6.90	6.84	6.85	6.79	0.05	0.61
NH_3_-N (mgN/dL)	0.82	0.73	0.84	0.93	0.96	0.07	0.22
Protozoa (×10^5^/mL)	0.94	0.86	0.67	1.00	0.93	0.11	0.34
DM degradability (%)	46.5^a^	46.5^a^	45.2^ab^	43.5^b^	45.4^ab^	0.45	< 0.01
VFA concentration							
Total VFA (mmol/L)	35.2	37.4	38.3	24.9	30.7	4.40	0.25
Acetate (%)	52.7	51.8	51.7	50.2	49.6	1.25	0.42
Propionate (%)	32.4^c^	33.9^b^	34.8^b^	35.6^a^	35.9^a^	0.23	< 0.01
iso-Butyrate (%)	0.2	0.3	0.0	0.0	0.0	0.17	0.55
Butyrate (%)	11.3	10.9	10.7	11.2	11.4	0.75	0.96
iso-Valerate (%)	1.5	1.4	1.3	1.1	1.4	0.30	0.87
Valerate (%)	1.9	1.6	1.6	2.0	1.9	0.24	0.79

^abc^LSMeans in a row with different superscripts significantly differ (P < 0.05).

SEM, standard error of means; DM, dry matter; NH_3_-N, ammonia nitrogen; VFA, volatile fatty acids

### Degradability, rumen fermentation characteristics, and protozoa population

No significant differences were observed in pH, NH_3_-N, or protozoa count among the treatments (Table 4). DM degradability in KJ2_L was lower than in CON (P < 0.05), whereas no significant differences were observed among the other treatments (Table 4). No significant differences were observed among the treatments in the total VFA concentration and proportion of each VFA except for propionate (Table 4). The proportion of propionate in the KJ1-supplemented treatments was higher than that in the CON treatment and was further increased in the KJ2-supplemented treatments (P < 0.05).

### Rumen microbiome after *in vitro* incubation

**Fig 3:**
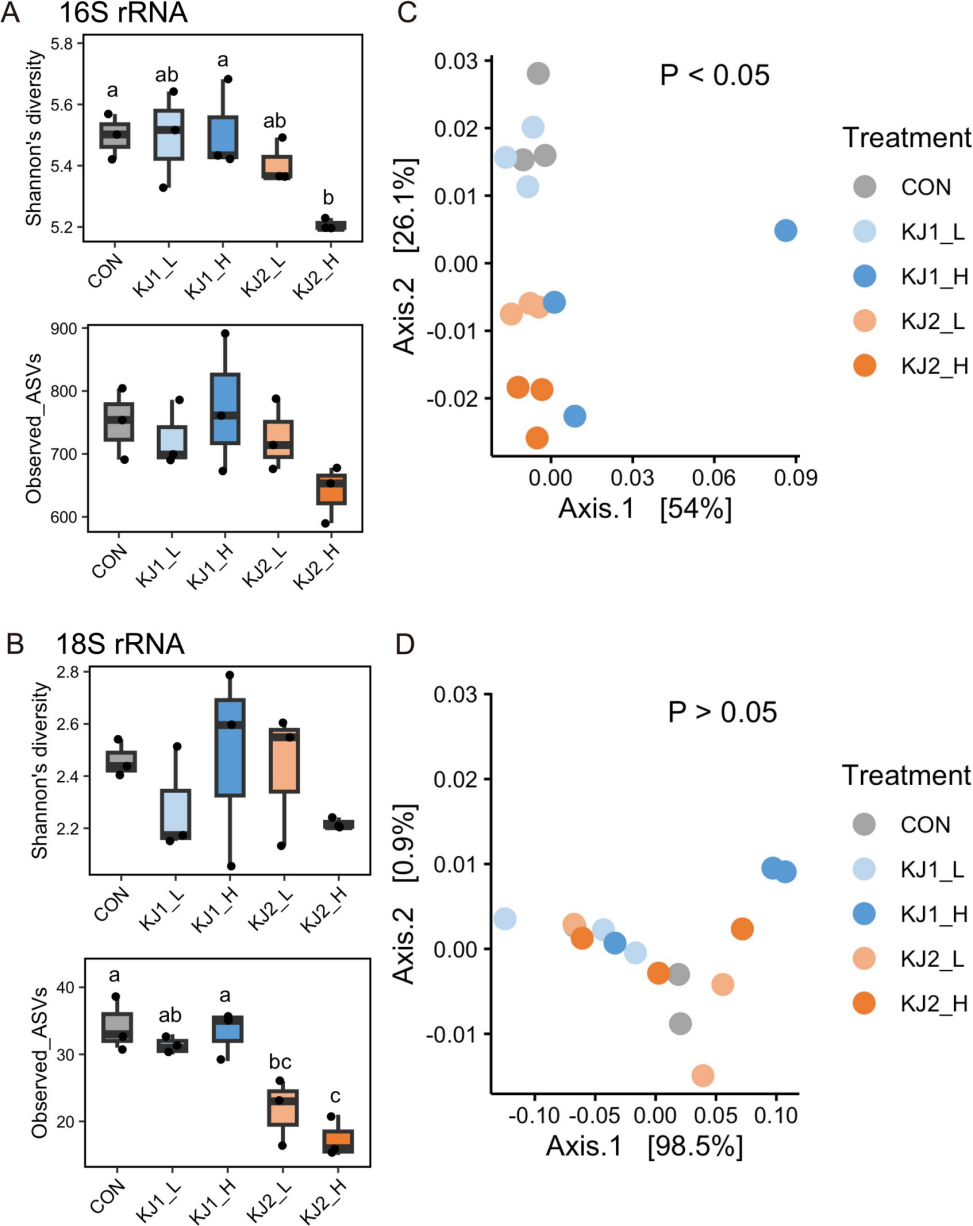
Rumen microbial diversity after 24 h of *in vitro* incubation. Alpha diversity of (A) rumen prokaryotes and (B) protozoa at the ASV level. Different letters at the top indicate significant differences between treatments (P < 0.05). Beta diversity of principal coordinate analysis (PCoA) based on weighted UniFrac distances of (C) rumen prokaryotes and (D) protozoa at the ASV level. Significance was analyzed using a permutational multivariate analysis of variance with 9,999 permutations.

The Shannon diversity index of KJ2_H was lower than that of CON for the rumen prokaryotes (Fig 3A). However, the number of observed ASVs was not significantly different among treatments (Fig 3A). Additionally, the observed ASVs of KJ2_L and KJ2_H were lower than those of CON for the rumen protozoa (Fig 3B). Beta diversity analysis based on weighted UniFrac distance showed significant differences in the rumen prokaryote communities among treatments (PERMANOVA, P < 0.05). In particular, prokaryotic communities in KJ2_L and KJ2_H were distinctly different from those in CON and KJ1_L (Fig 3C). However, no differences were observed among treatments for rumen protozoa communities (Fig 3D).

**Fig 4:**
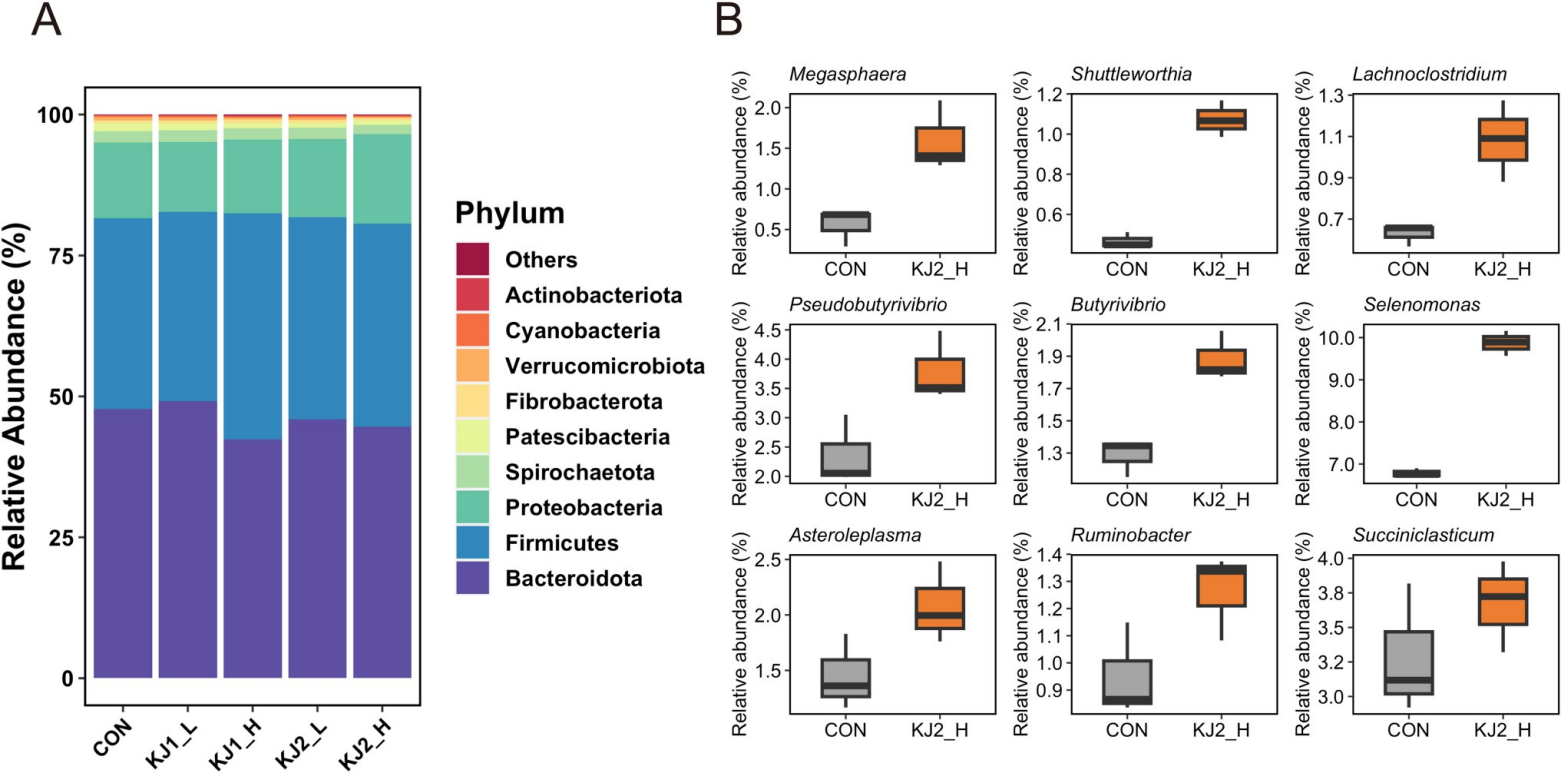
Microbial communities after 24 h of *in vitro* incubation. (A) Taxonomic distribution of the rumen microbiome at the phylum level. All phyla with a relative abundance of < 0.1% in all treatments were combined into “Others.” (B) Relative abundances of significantly different genera between CON and KJ2_H (adjusted P < 0.05). Differential genera were identified using DESeq2. Only genera with a relative abundance of at least 0.5% were present.

Based on 16S rRNA amplicon sequencing, 18 bacterial and two archaeal phyla were observed, and Bacteroidota, Firmicutes, and Proteobacteria accounted for approximately 95% of the total abundance (Fig 4A). Compared to CON, the abundance of Verrucomicrobiota and Patescibacteria was low while that of Firmicutes was high in KJ1_H and KJ2_H (adjusted P < 0.05) (S1 Table). Additionally, the abundances of 14, 11, and 22 genera were significantly different in KJ1_H, KJ2_L, and KJ2_H, respectively, compared with CON (S1 Table). However, no significant difference was observed between CON and KJ1_L (S1 Table). Compared with CON, *Butyrivibrio*, *Shuttleworthia*, *Megasphaera*, and *Succiniclasticum* were enriched in KJ1_H and KJ2_H (adjusted P < 0.05) (Fig 4B and S1 Table). Furthermore, the abundance of *Pseudobutyrivibrio*, *Selenomonas*, and *Ruminobacter* was also higher in KJ2_H than in CON (adjusted P < 0.05) (Fig 4B and S1 Table). In contrast, the abundance of 10 genera, most of which accounted for < 1.0% of total abundance, was lower in KJ2_H than in CON (adjusted P < 0.05).

For the rumen protozoa, only one phylum, Ciliophora, was identified. Seven genera were identified, and *Entodinium* was the most abundant (93.5%), followed by *Charonina* (2.4%), and *Diplodinium* (2.3%) (S2 Fig). Only *Polyplastron* in KJ2_H was lower than that in CON (adjusted P < 0.05); however, no significant differences were observed at the genus level between CON and the other treatments.

## Discussion

To our knowledge, this study is the first to investigate the effects of two types of *Coccomyxa* sp. KJ (KJ1 and KJ2) on rumen total gas and CH_4_ production, rumen fermentation characteristics, and the rumen microbiome under *in vitro* conditions. The use of microalgae as feed additives has been predicted to inhibit CH_4_ production from the rumen. In a previous study, adding 10 and 25% *E*. *gracilis* reduced ruminal CH_4_ production *in vitro* by 4.4 and 11%, respectively, when hay and concentrate (50%:50%) were used as substrates [17]. Additionally, *in vitro* ruminal CH_4_ production was reduced by approximately 19% by supplementation with 2% and 3% *C*. *vulgaris* [18]. In this study, supplementary KJ1 and KJ2 decreased CH_4_ production by 34.1–51.3% compared to that in CON, indicating that *Coccomyxa* sp. KJ, particularly KJ2, had a significantly stronger inhibitory effect on ruminal CH_4_ than other microalgae. Importantly, the amounts of additives used in this study were very low, from 0.5 to 1.0%. Generally, feed additives are more expensive than basal diet. Therefore, the addition of a significantly lower amount of *Coccomyxa* sp. KJ is an economical and feasible strategy to decrease ruminal CH_4_ production.

One possible factor for reducing ruminal CH_4_ is the fatty acid content, particularly monounsaturated fatty acids (MUFAs) and PUFAs, in *Coccomyxa* sp. KJ. In a previous study, Martin et al. [7] reported that adding 5.7% linseed oil, which has a high PUFA content, inhibited CH_4_ production from dairy cows by 64%. Furthermore, calcium salts of long-chain fatty acids, most of which are PUFAs, from linseed oil drastically decrease *in vitro* ruminal CH_4_ production [8, 9]. KJ1 and KJ2 used in this study included high amounts of oleic and linolenic acid, respectively. Considering that the ruminal CH_4_ reduction effect of linolenic acid is higher than that of oleic acid [45], KJ2 likely had a greater reduction effect on ruminal CH_4_ production than KJ1. However, it is curious that *Coccomyxa* sp. KJ had a significant reduction effect on ruminal CH_4_ emissions, although the ether extract content of KJ1 and KJ2 was only 11.5–22.8% DM, and the amount of fatty acids added was much lower than that in previous studies [7, 8]. Therefore, substances other than fatty acids in *Coccomyxa* sp. KJ may be responsible for inhibiting ruminal CH_4_.

Although CH_4_ synthesized by methanogens using H_2_ and CO_2_ as substrates is the primary H_2_ sink in the rumen, propionate production is associated with disposable H_2_ [46]. Therefore, increasing the proportion of propionate competes for H_2_ with methanogenesis by methanogens, thereby inhibiting CH_4_ production. Several studies have demonstrated that the proportion of propionate in the rumen increases with CH_4_ inhibition [8, 47, 48]. Similarly, compared with the CON treatment, the addition of KJ1 increased the proportion of propionate, and the addition of KJ2 led to an even greater increase in the current study.

The increase in propionate may be attributed to changes in the rumen microbiome caused by the addition of KJ1 and KJ2. Beta diversity analysis indicated that the ruminal microbiota in KJ1_H, KJ2_L, and KJ2_H was significantly different from that in CON. In addition, the proportion of some bacterial genera related to propionate production increased with the addition of 1.0% KJ2. For example, when 1.0% KJ2 was added, a significant increase was observed in the relative abundances of *Selenomonas*, *Succiniclasticum*, and *Ruminobacter*, which are associated with propionate synthesis via the succinate pathway. This result is consistent with that of a previous study in which ruminal CH_4_ was inhibited after adding calcium salts of long-chain fatty acids [8]. *Ruminobacter* produces succinate in the rumen [49], whereas *Selenomonas* and *Succiniclasticum* can promote the metabolism of carbohydrate fermentation-derived succinate to propionate [50, 51]. Furthermore, *Megasphaera*, which converts lactate to propionate via the acrylate pathway in the rumen [49], was also enriched in the KJ2_H treatment. *Megasphaera* spp. are more abundant in low than in high-CH_4_-emitting sheep [52], and *Megasphaera elsdenii* is more abundant in the rumen of cows with a high feed efficiency [53]. The relative abundance of the genus *Megasphaera* is positively correlated with the average daily gain [54] and microbial proteins that can be used to synthesize milk proteins [55]. Therefore, increasing *Megasphaera* abundance by adding KJ2 would benefit milk production. The abundance of *Shuttleworthia*, which is positively correlated with the propionate concentration in the rumen [56, 57], was also increased in KJ1_H, KJ2_L, and KJ2_H. Thus, the increasing proportion of propionate produced through the addition of KJ1 and KJ2 can be attributed to the higher abundance of these genera that contribute to propionate production.

Similarly, the abundance of *Butyrivibrio* and *Pseudobutyrivibrio*, the main butyrate-producing bacteria in the rumen [58], increased with the addition of 1.0% KJ2. Some researchers have demonstrated that the abundance of these genera is positively correlated with CH_4_ emissions [59], which is inconsistent with our results. As *Butyrivibrio* spp. and *Pseudobutyrivibrio* spp. can perform ruminal biohydrogenation of unsaturated fatty acids, such as linoleic and α-linolenic acid [60–63], increased PUFAs caused by adding KJ2 lead to an increase in the abundance of these bacteria. Thus, *Butyrivibrio* spp. and *Pseudobutyrivibrio* spp. may not be positively correlated with CH_4_ emissions when PUFA suppress CH_4_ production in the rumen.

Ciliate protozoa, which are hydrogen producers in the rumen, harbor methanogens on the cell surface and in the cytoplasm as endosymbionts [64, 65]. Interspecies hydrogen transfer has been observed between rumen ciliates and methanogens, resulting in enhanced methanogenesis in the rumen [66]. In the present study, the alpha diversity of protozoa decreased in the 0.5% and 1.0% KJ2 treatments compared to the CON treatment, suggesting that KJ2 has a toxic effect against protozoa. The reduction in protozoan diversity may be related to suppressing ruminal CH_4_ production.

The addition of KJ1 and KJ2 did not affect ruminal pH, NH_3_-N concentrations, and total VFAs, suggesting that *Coccomyxa* sp. KJ had no negative effect on ruminal characteristics. However, a slight reduction in DM degradability was observed with the addition of 0.5% KJ2 (CON: 46.5%; KJ2_L: 43.5%). This finding may be attributed to the antimicrobial effect of *Coccomyxa* sp. KJ, particularly MUFAs and PUFAs, on bacteria. In the present study, reduced prokaryotic alpha diversity was observed after addition of 1.0% KJ2. Similarly, the addition of calcium salts of long-chain fatty acids decreases alpha diversity, resulting in reduced DM degradability and CH_4_ production [8].

In this study, we evaluated the effects of supplementation with *Coccomyxa* sp. KJ as the corn silage source because corn silage is a common roughage source in dairy production. Although many studies have investigated the effects of feed additives on *in vitro* rumen fermentation under corn silage conditions [67, 68], different effects have been reported for substrate at different concentrate to roughage ratios [69]. Therefore, we need to verify whether supplementary *Coccomyxa* sp. KJ has an inhibitory effect on ruminal CH_4_ under different substrate conditions.

In conclusion, both types of *Coccomyxa* sp. KJ—KJ1 and KJ2—had a high inhibitory effect on rumen CH_4_ production when only 0.5–1.0% was added. In particular, KJ2 had a greater inhibitory effect on ruminal CH_4_ production than KJ1. Furthermore, *Coccomyxa* sp. KJ modified the rumen microbiome, resulting in increased propionate and decreased CH_4_ production. These findings provide important information for inhibiting ruminal CH_4_ emissions, which is essential for increasing animal productivity and sustaining livestock production under future population growth. Future *in vivo* studies are needed to validate the inhibitory effect and to determine the optimal dose of *Coccomyxa* sp. KJ supplementation without decreasing digestibility and productivity.

## Supporting information

S1 Fig *In vitro* gas production during 24 h of incubation. (TIF)

S2 Fig Relative abundance of protozoa after 24 h of incubation. (TIF)

S1 Table Relative abundance (%) of each taxa at phylum and genus level in treatment. ^1^Taxa with significant differences between CON and other treatments (adjusted P < 0.05). ^2^Treatments with higher or lower abundance compared to CON.
(XLSX)
