# Supplementary figures and images for: Effects of two types of Coccomyxa sp. KJ on in vitro ruminal fermentation, methane production, and the rumen microbiota
Source: PLoS One. 2024 Aug 22;19(8):e0308646. doi: 10.1371/journal.pone.0308646 (PMC11341058; doi:10.1371/journal.pone.0308646)

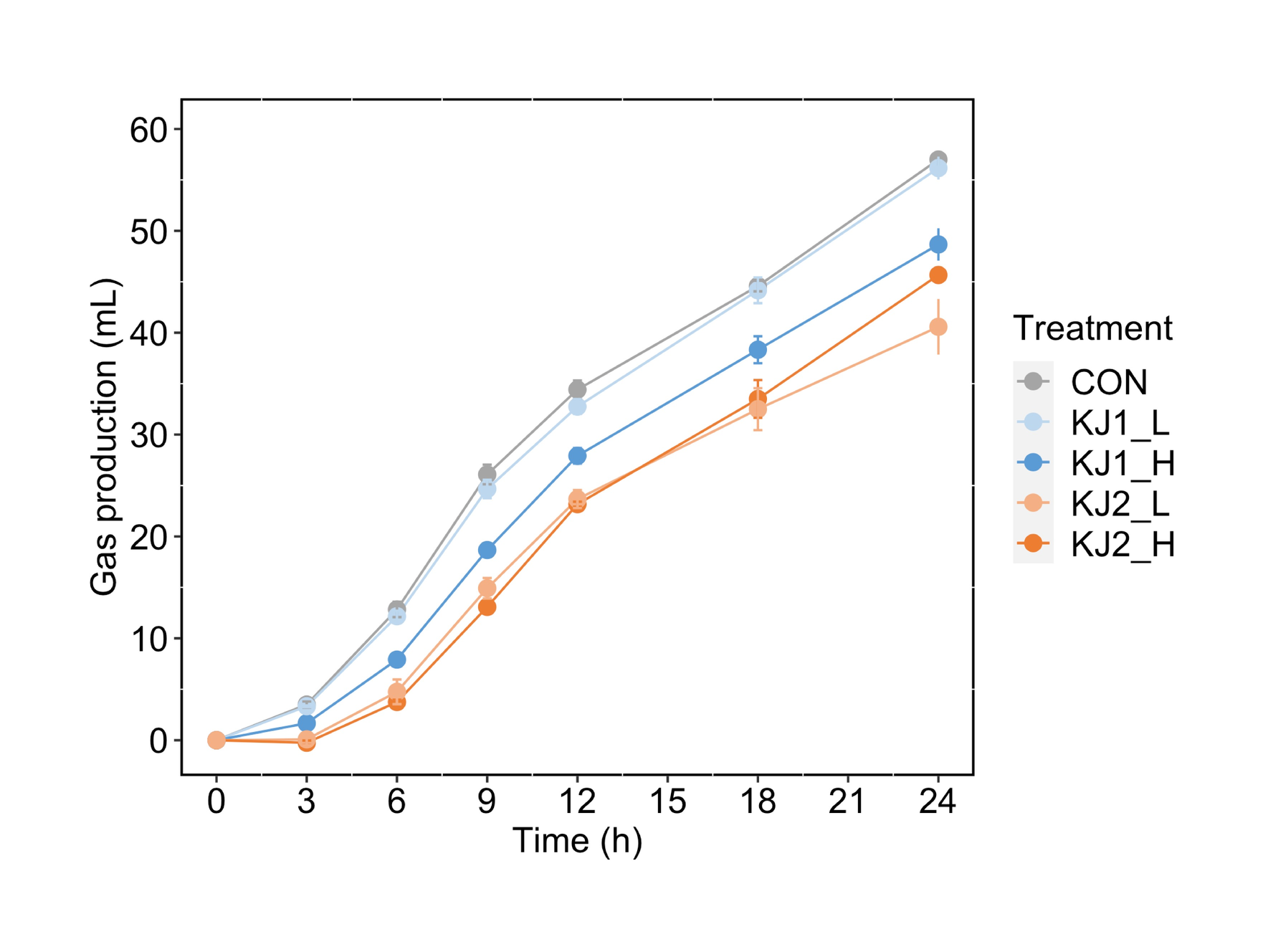

Supplement: S1 Fig — (TIF) [file pone.0308646.s001.tif]

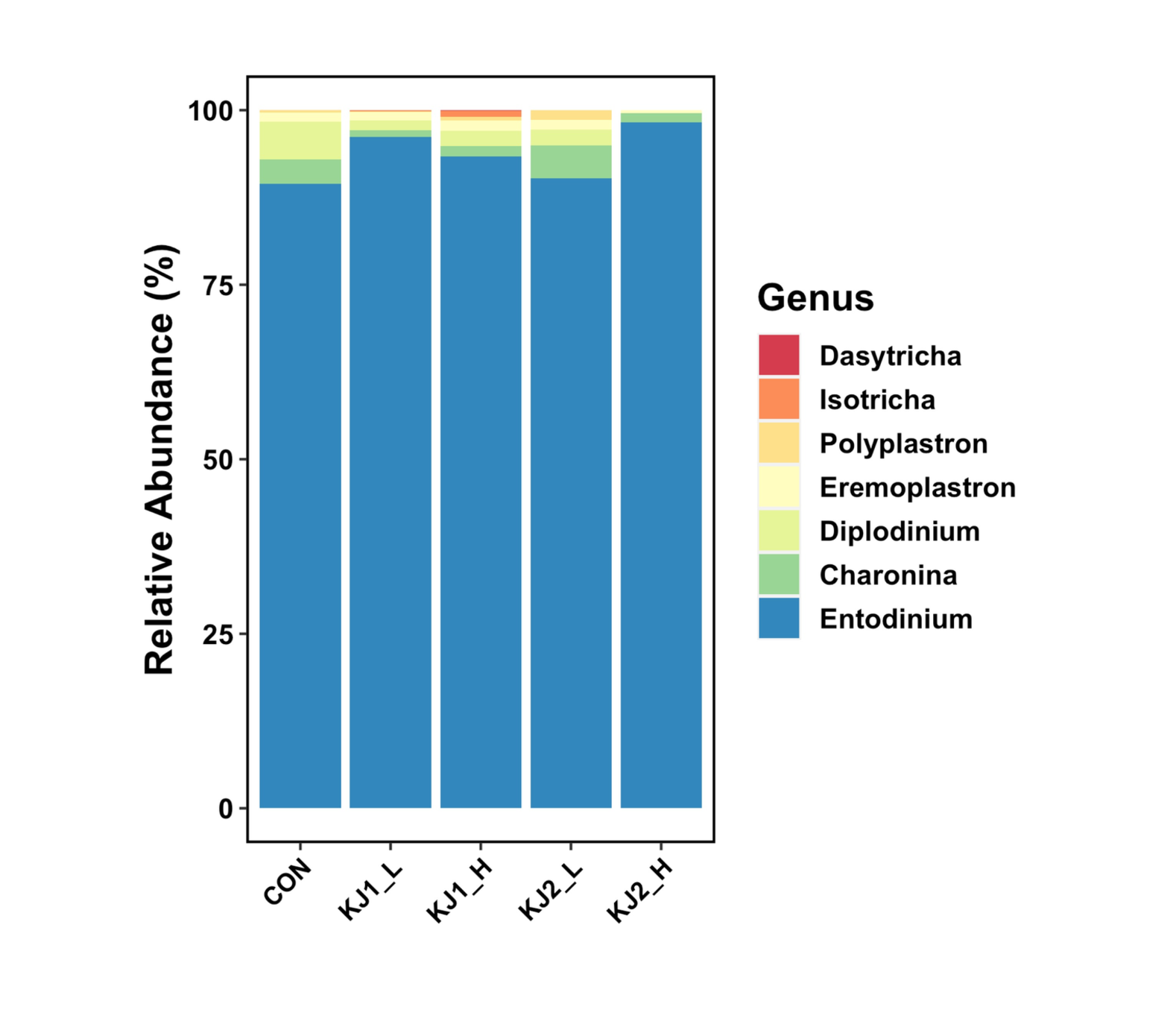

Supplement: S2 Fig — (TIF) [file pone.0308646.s002.tif]
